# Physiological and Mechanical Responses to a Graded Exercise Test in Traditional Rowing

**DOI:** 10.3390/ijerph20043664

**Published:** 2023-02-18

**Authors:** Alfonso Penichet-Tomas, Jose M. Jimenez-Olmedo, Basilio Pueo, Javier Olaya-Cuartero

**Affiliations:** Research Group in Health, Physical Activity, and Sports Technology (Health-Tech), Faculty of Education, University of Alicante, 03690 San Vicente del Raspeig, Spain

**Keywords:** exercise physiology, endurance performance, maxima aerobic power, anthropometry, heart rate, rowing ergometer, fixed seat rowing

## Abstract

Maximum oxygen consumption and maximum power output are critical measures for training prescription in endurance sports such as rowing. The objective of this investigation was twofold: to compare the physiological and mechanical responses of female and male traditional rowers during a graded exercise test and to establish reference values in this specific rowing modality that have not yet been documented, unlike in Olympic rowing. Twenty-one highly trained/national level rowers participated in the study: 11 female (age: 30.1 ± 10.6 years, height: 167.3 ± 5.0 cm, body mass: 61.9 ± 4.9 kg) and 10 males (age: 33.5 ± 6.6 years, height: 180.8 ± 6.9 cm, body mass: 74.4 ± 6.9 kg). Significant differences (*p* < 0.05) were found in rowing performance between sexes, with a very large effect size (*d* = 7.2). The peak power output for the female rowers was 180.9 ± 11.4 W and 287.0 ± 17.7 W for the male rowers. The female rowers reached a VO_2max_ of 51.2 ± 6.6 mL/kg/min at a mean of 174.5 ± 12.9 W, while the males’ VO_2max_ was 62.1 ± 4.7 mL/kg/min at a mean of 280.0 ± 20.5 W. These differences in VO_2max_ and maximal aerobic capacity were significant (*p* < 0.05), with a large (*d* = 1.9) and very large (*d* = 6.2) effect size, respectively. A moderate association between VO_2max,_ and rowing performance expressed in watts per kilogram of muscle mass was observed in the female rowers (*r* = 0.40, *p* = 0.228). For the male rowers, the correlation between VO_2max_ and relative peak power output in watts per kilogram of body mass was strong (*r* = 0.68; *p* = 0.031). This study highlights the differences in the kinetics of ventilatory and mechanical parameters between female and male rowers and the importance of these differences for specific physical preparation in traditional rowing.

## 1. Introduction

Laboratory exercise testing is a crucial tool for personalized training prescription in endurance athletes, as well as for monitoring progress and evaluating performance [1]. Performance evaluation is significant for athletes, coaches, and trainers, especially if the results are translated into recommendations for daily training [2]. These tests enable the assessment of aerobic capacity and customization of exercise prescriptions according to the specific profile of the sport discipline and the athlete [3]. The data gathered from exercise testing offer valuable insights into physiological characteristics and adaptations to controlled exercise loads [4]. They can even be used for talent selection and scouting for potential future champions [5].

Gas exchange measurements during exercise testing have served as the primary assessment tool for decades, though they were overshadowed by the refinement of lactate diagnostics during the 1970s and early ’80s. However, with the new methodological improvements in their measurements [6], they have regained significance, and maximal oxygen uptake (VO_2max_) and maximal power output achieved during exercise testing are the most frequently applied indicators of endurance capacity [7,8,9]. VO_2max_ reflects the body’s ability to absorb, transport, and metabolize oxygen and is dependent not only on the oxygen metabolism of the muscle to generate energy but also on the respiratory system that absorbs oxygen from the air, the cardiovascular system responsible for delivering it to the muscles, and the blood and haemoglobin that carry it [10,11]. Furthermore, it is considered the most important indicator of endurance capacity in sports science [9].

The combination of high endurance and aerobic capacity and overall muscular strength [12] allows rowers to produce maximal oxygen consumption [13] during the 5.5 to 7.0 min it takes to cover a distance of 2000 m in a straight line [14]. This results in high absolute VO_2max_ values, with male rowers reaching 7 L/min and female rowers reaching 5.5 L/min [11]. Aerobic metabolism provides a significant portion, 75% to 80%, of the energy needed in a rowing competition, highlighting the importance of aerobic performance in the sport [15]. On the other hand, anaerobic metabolism contributes between 12% and 30% [16].

However, the physiological responses vary among athletes and are influenced not only by factors such as gender or training level [17], but also by the different modalities of the same sport [18]. There are two main types of rowing: Olympic rowing, which involves a moving seat, and traditional rowing, which utilizes a fixed seat. In Olympic rowing, the seat moves stern and bow, while in traditional rowing there is no seat movement. In the latter modality, the rower’s support of the ischial region is located on a fixed bench instead of sitting on a mobile seat [19]. This modality is called traditional rowing because it is how rowing was originally practiced. The difference in the type of seat means that the legs contribute 46%, the trunk 32% and the arms 22% of the power required for each stroke in Olympic rowing [20]. However, in traditional rowing, the legs provide about 40% and the trunk and arms contribute the remaining 60% [18]. Traditional rowing courses in Llaüt are held in four lengths with three complete turns over 1400 m of total distance and last for a similar average time to Olympic rowing. Although these technical and competitive differences may result in varying functional and physiological demands, there is no scientific evidence to support this.

There are also differences in the physiological responses between male and female athletes. VO_2max_ can be measured in absolute (L/min) and relative (mL/kg/min) terms. Elite female rowers generally have 10% lower relative values of VO_2max_ compare to male rowers, likely due to higher body fat levels and lower red cell mass per body weight in women [21]. The VO_2max_ ever recorded was 94 mL/kg/min for males and 77 mL/kg/min for females, both cross-country skiers [22]. Despite these differences, heavier female rowers appear to achieve higher absolute VO_2max_ values than those observed in shorter male long-distance runners [23]. In line with the differences in anthropometric variables and body composition, VO_2max_ expressed per kilogram of fat-free mass could also be comparable in elite endurance athletes. In fact, high values of body mass and height were associated with rowing performance inin male and female traditional rowers. The best predictors of rowing performance for male and female rowers were height and muscle mass, respectively [24,25]. This suggests that there may be differences in some of the physiological determinants of VO_2max_ in females compared to males.

VO_2max_ is a measure of the body’s oxygen uptake capacity during physical activity and can be measured through a graded exercise test (GXT) using a metabolic gas measurement system. GXT is one of the most common methods used to assess the aerobic capacity of athletes in laboratory conditions [26,27]. Compared to step tests, a power increase in shorter intervals does not expose the athlete to such abrupt changes in intensity, allowing a more appropriate physiological assessment [28]. As exercise intensity increases, so does oxygen consumption, but a point is reached at which exercise intensity can continue to increase without a corresponding increase in oxygen consumption [29]. However, the assessment of VO_2max_ on water is affected by external factors, such as weather and water conditions, making it unreliable [30]. The use of rowing ergometers, which simulate the rowing experience and provide a controllable and repeatable environment [1,14,31], is considered the gold standard for directly measuring VO_2max_ in a laboratory setting [9].

The objective of this investigation was twofold: to compare the physiological and mechanical responses of female and male traditional rowers during a graded exercise test and to establish reference values in this specific rowing modality that have not yet been documented, unlike in Olympic rowing. Based on previous research on Olympic rowing and gender-based performance differences, it was hypothesized that significant differences would exist between male and female traditional rowers.

## 2. Materials and Methods

### 2.1. Experimental Design

A cross-sectional observational study was conducted to compare physiological responses in a performance rowing test between female and male rowers. The study design included a two-day design that comprised an anthropometric evaluation and a Graded Exercise Test (GXT) on a rowing ergometer. On the day of the anthropometric evaluation, rowers were instructed to refrain from eating for at least four hours before the measurements, not exercise on the day of the measurement [24,32], and not high-intensity exercise on the day before. On the day of the GXT, rowers were advised to avoid high-intensity physical activity in the previous 48 h [27], and not to consume any type of stimulant for at least 5 h before testing [19]. All tests were performed in a temperature-controlled laboratory (temperature 18 °C to 23 °C, humidity < 70%) [30,33].

### 2.2. Participants

Twenty-one highly trained/national level rowers participated in the study [34]: 11 female (age: 30.1 ± 10.6 years, height: 167.3 ± 5.0 cm, body mass: 61.9 ± 4.9 kg, muscle mass: 39.3 ± 2.7%, fat mass: 14.0 ± 2.1%, VO_2max_: 51.2 ± 6.6 mL/kg/min) and 10 males (age: 33.5 ± 6.6 years, height: 180.8 ± 6.9 cm, body mass: 74.4 ± 6.9 kg, muscle mass: 43.2 ± 1.4%, fat mass: 9.0 ± 2.3%, VO_2max_: 62.1 ± 4.7 mL/kg/min). The requisites to participate were to have an experience of at least 3 consecutive full years, not to have any musculoskeletal or neurological disorders, and to regularly train of six days per week for 2 h/day. Rowers who did not meet the selection criteria were excluded from the study. Prior to applying the evaluation protocols, all participants gave their written consent after the project information, which was previously approved by the research ethics committee of the University of Alicante (IRB No. UA-2010-07-21).

### 2.3. Antropometric Measurements and Body Composition

The anthropometric evaluation followed the guidelines established by the International Society for the Advancement of Kinanthropometry (ISAK) [35]. All variables were measured twice on the right side of the body, and the mean value was recorded. Body mass and height were measured using a scale (model 707, Seca, Hamburg, Germany) to the nearest 0.1 kg and a stadiometer (Harpenden, Burgess Hill, UK) to the nearest 0.1 cm. The rower’s height was measured while they were upright and their chin was parallel with the ground. Body mass index (BMI) was computed as body mass (kg) divided by height squared (m^2^). To calculate the percentage of fat mass, the formula of Withers et al. was used [36]. Muscle mass was determined using the methods of Lee et al. [37].

### 2.4. Graded Exercise Test (GXT)

GXT was performed on a rowing ergometer (Concept 2, Model D, monitor PM5, Morrisville, VT, USA) with a fixed seat adapter, reducing the range of motion of the legs to reproduce the biomechanics of traditional rowing stroke [24,38]. The drag factor was set at 145 [39,40] for male rowers and 135 for female rowers [41]. The rowers completed a standardized 10 min warm-up at continuous intensity [30] with heart rate below 140 beats per minute and 18–20 strokes per minute [25].

The GXT protocol consisted of 1 min exercise stages without rest [30,42]. The initial workload was 100 W [38,42,43] for male rowers and 60 W for female rowers [33]. The increments were 10 W for each stage instead of 30 W every 3 min, with 1 min rests [1], due to the lack of lactate sample collection. Compared to step tests, a power increase in shorter intervals does not expose the athlete to such abrupt changes in intensity, allowing a more appropriate physiological assessment [28]. The rowers were instructed to maintain an average power output as close as possible for each stage [1]. The test was automatically ended if a rower failed to increase the mechanical power output within a 7 W range of five strokes [44]. Peak power output (PPO) was defined as the highest stage the subject successfully completed. PPO, measured in watts, and relative PPO, calculated in watts per kilogram and watts per muscle mass kilogram, were assessed as indicators of rowing performance [31,45].

### 2.5. Maximum Oxygen Uptake

The validated portable metabolic analyser VO_2_ Master Pro (VO_2_ Master Health Sensors Inc., Vernon, BC, Canada) was used to measure cardiorespiratory parameters [46]. Before each trial, the analyser was calibrated according to the manufacturer’s instructions. The analyser was connected via Bluetooth to the PM5 performance monitor of the rowing machine to monitor and collect power output, and with an H7 Bluetooth Smart Band (Polar Inc., Kempele, Finland) [19,47] to monitor and collect heart rate. VO_2max_ was defined as the highest 30 s moving average and considered maximum if VO_2_ failed to increase with progressive work rate or at least a plateau was observed [48]. Following Howley et al. [49] criteria, it was also verified that HR reached within 10 beats of the age-adjusted HR_max_ upon using the 220-age equation. Maximal aerobic power was set at the intensity associated with VO_2max_ [50].

### 2.6. Statistical Analysis

Statistical analyses were performed using the Statistical Package for Social Sciences (SPSS v.26 for Windows, SPSS Inc., Chicago, IL, USA). Descriptive analysis was shown with the mean and standard deviation (SD) for all variables, with 95% confidence intervals (CI) via bootstrapping. Data normality was confirmed by Shapiro–Wilk tests. Student’s *t*-test was used to compare the means of the parameters between female and male rowers. Cohen’s *d* was used as a measure of the effect size of differences between female and male rowers and interpreted according to modified thresholds [51] for sports sciences [52] as trivial (<0.2), small (0.21–0.6), moderate (0.61–1.2), large (1.21–1.99), and very large (>2.0). The Pearson correlation coefficient (*r*) was used to establish relationships between VO_2max_ and rowing performance. The magnitude of the correlation coefficient was interpreted with the following thresholds: trivial (<0.1), small (0.1–0.29), moderate (0.3–0.49), strong (0.5–0.69), very strong (0.7–0.89), nearly perfect (0.9–0.99), and perfect (1.0) [52]. Statistical significance was set at *p* < 0.05.

## 3. Results

### 3.1. Anthropometric Characteristics

Table 1 shows the results of the anthropometric measures and the independent samples *t*-test between female and male traditional rowers. The male rowers had larger values of anthropometric characteristics and muscle mass (33.4 ± 1.9 kg vs. 24.3 ± 1.7 kg) compared to the female rowers. These differences were statistically significant (*p* < 0.05) with moderate to very large effect size values. However, the female rowers showed higher values in fat mass (14.0 ± 2.1% vs. 9.0 ± 2.3%), with significant differences (*p* < 0.05) in the percentage of fat mass, with a very large effect size (*d* = 2.3). However, there were no significant differences found in kilograms of fat mass (*p* = 0.063).

### 3.2. Graded Exercise Test

Peak power output was considered a performance indicator, defined as the highest stage the subject successfully completed at GXT. Table 1 shows that peak power output (287.0 ± 17.7 W in male and 180.9 ± 11.4 W in female rowers), and relative peak power output, both per kilogram of body mass and per kilogram of muscle mass, were higher in males than in females. These differences were statistically significant (*p* < 0.05) and had a very large effect size. However, the female rowers needed a higher maximal stroke rate (37.9 ± 2.9 spm) than the male rowers (33.9 ± 1.6 spm). This difference was statistically significant (*p* < 0.05) with a large effect size (*d* = 1.7). Although the women reached a lower maximum heart rate, there were no statistically significant differences.

**Table 1:** Mean values of anthropometric characteristics and GXT parameters. Difference between female and male rowers.

	Female (*n* = 11)		Male (*n* = 10)		*p*	Effect Size	
	Mean ± SD	95% CI	Mean ± SD	95% CI		*d*	Size
Anthropometry							
Height c(m)	167.3 ± 5.0	164.3–170.3	180.8 ± 6.9	175.5–184.0	<0.001 *	1.0	Moderate
Body Mass (kg)	61.9 ± 4.9	59.1–64.4	74.4 ± 6.9	70.5–78.5	<0.001 *	2.1	Very Large
BMI (kg/m^2^)	22.1 ± 1.8	21.1–23.1	23.0 ± 1.1	22.3–23.6	0.207	0.6	Small
Muscle Mass (%)	39.3 ± 2.7	37.9–40.8	43.2 ± 1.4	42.2–43.9	0.001 *	1.8	Large
Fat Mass (%)	14.0 ± 2.1	12.8–15.2	9.0 ± 2.3	7.7–10.3	<0.001 *	2.3	Very Large
Muscle Mass (kg)	24.3 ± 1.7	23.4–25.4	33.4 ± 1.9	32.3–34.5	<0.001 *	5.1	Very Large
Fat Mass (kg)	8.7 ± 1.7	7.7–9.7	7.0 ± 2.2	5.8–8.4	0.063	0.9	Moderate
Graded Exercise Test							
PPO (W)	180.9 ± 11.4	175.5–187.3	287.0 ± 17.7	277.0–298.0	<0.001 *	7.2	Very Large
PPO (W/kg)	2.9 ± 0.3	2.8–3.1	3.9 ± 0.3	3.7–4.0	<0.001 *	3.3	Very Large
PPO (W/MMkg)	7.5 ± 0.5	7.2–7.7	8.6 ± 0.4	8.4–8.8	<0.001 *	2.4	Very Large
Time (s)	785.5 ± 68.2	752.7–823.6	1182.0 ± 106.0	1122.0–1248.0	<0.001 *	4.5	Very Large
HR_max_ (bpm)	182.6 ± 8.6	177.5–187.2	187.9 ± 7.0	182.9–191.6	0.104	0.7	Moderate
SR_max_ (spm)	37.9 ± 2.9	36.3–39.5	33.9 ± 1.6	33.0–34.8	0.001 *	1.7	Large

SD: standard deviation; CI: confidence interval; BMI: body mass index; PPO: peak power output; MMkg: muscle mass in kilograms; HR_max_: maximal heart rate; bpm: beat per minute; SR_max_: maximal stroke rate; spm: stroke per minute; *: statistically significance between female and male rowers (*p* < 0.05).

### 3.3. VO_2max_, Ventilatory and Mechanical Parameters

The comparative analysis presented in Table 2 shows that the female rowers reached a VO_2max_ of 51.2 ± 6.6 mL/kg/min at a mean of 174.5 ± 12.9 W with 180.7 ± 9.1 bpm. Absolute VO_2max_ was 3.2 ± 0.5 L/min. Meanwhile, the male rowers achieved a VO_2max_ of 62.1 ± 4.7 mL/kg/min at a mean of 280.0 ± 20.5 W with 185.9 ± 7.0 bpm. Absolute VO_2max_ was 4.7 ± 0.5 l/min. The male rowers showed higher VO_2max_ and power output at VO_2max_ than the female rowers, with significant differences (*p* < 0.05) and large to very large effect size values. However, no significant differences were found in HR and SR at VO_2max_ between the female and male rowers.

**Figure 1:**
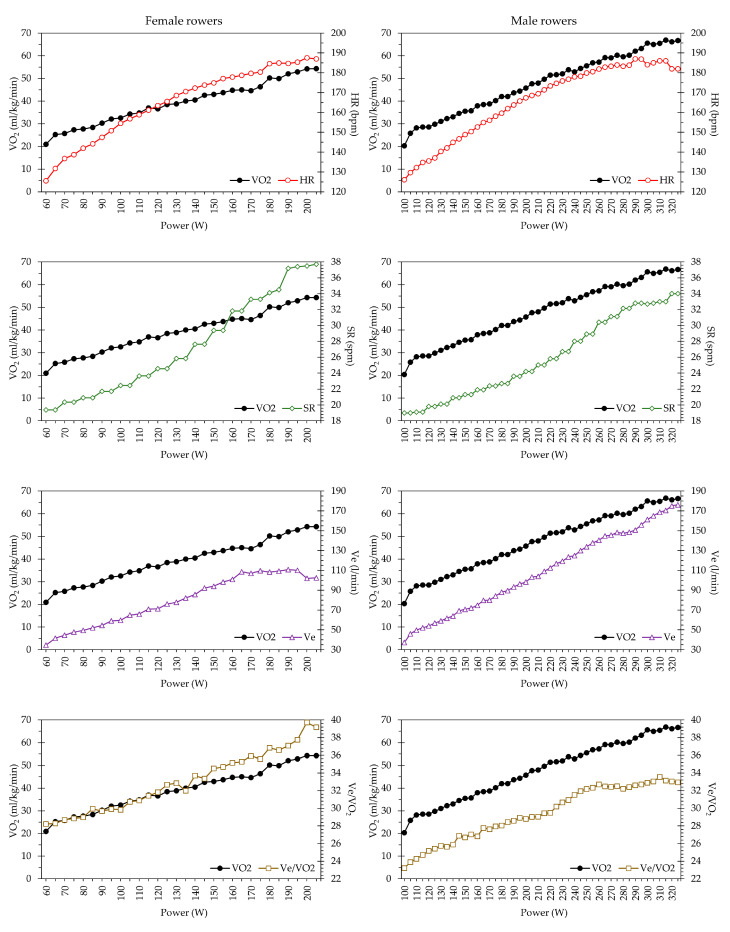
Kinetics of metabolic, mechanical, ventilator, and gas exchange parameters in a grade exercise test on a rowing ergometer.

Figure 1 displays the HR kinetics for both female and male rowers, which exhibit a linear trend at submaximal intensities and flatten as the maximum intensity is approached. No significant differences between the sexes (*p* = 0.163) were found. Although the SR of the female rowers was higher, especially at the end, no significant difference was found (*p* = 0.074). Regarding the ventilatory parameters, Ve at VO_2max_ of male rowers was 156.0 ± 17.2 l/min with a Tv of 2.6 ± 0.3 l. However, the Ve of the female rowers was 115.6 ± 11.9 L/min with a Tv of 2.0 ± 0.2 l. These differences were statistically significant (*p* < 0.05) with a very large effect size. Figure 1 shows how the Ve flattens out from 170 W in the female rowers, while in the male rowers it increases steadily until the end of the test, except for a small plateau at 270 W. There were no significant differences either in respiratory frequency (Rf) or gas exchange, where Ve/VO_2_ was higher in the female than in the male rowers, starting with lower values and ending with higher values.

### 3.4. VO_2max_ and Performance in Rowing: Absolute and Relative Values

**Figure 2:**
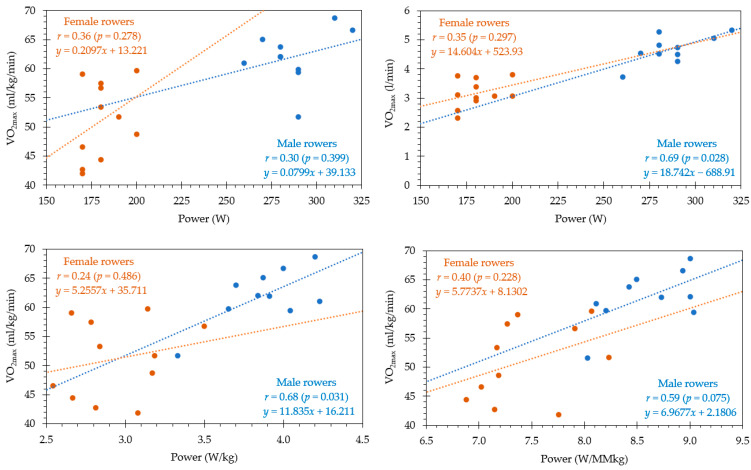
Relationships between VO_2max_ and rowing performance in female and male rowers.

Figure 2 shows the relationship between VO_2max,_ and rowing performance expressed in peak power output reached at GXT. The results show a moderate correlation between VO_2max_ in mL/kg/min with rowing performance in the female (*r* = 0.36; *p* = 0.278) and male rowers (*r* = 0.30; *p* = 0.399). However, the results show a similar association between VO_2max_ in L/min with rowing performance in the female rowers (*r* = 0.35; *p* = 0.297) but a strong correlation in the male rowers (*r* = 0.69; *p* = 0.028).

The relative peak power output In watts per kilogram of body mass can be considered a performance variable in rowing. In the female rowers, the association with VO_2max_ decreases (*r* = 0.24; *p* = 0.486), while the association in the male rowers remains strong (*r* = 0.68; *p* = 0.031). Finally, it should be noted that the highest correlation in the female rowers is shown between VO_2max_ and relative peak power output in watts per kilogram of muscle mass (*r* = 0.40; *p* = 0.228), although it remains a moderate correlation. However, in the male rowers, it reaches the lower values of strong correlation (*r* = 0.59; *p* = 0.075).

## 4. Discussion

The main aim of this study was to compare the physiological and mechanical responses during a GXT test performed by female and male traditional rowers and to establish reference values in this specific rowing modality that have not yet been documented, unlike in Olympic rowing. There are currently no data available on the kinetics of these parameters in traditional rowing, nor any association between maximum oxygen uptake and rowing performance. It was hypothesized that significant differences would exist between female and male traditional rowers, based on previous studies on Olympic rowing and observed differences in performance indicators between the sexes in traditional rowing.

The results of this study indicate that male rowers have a significantly higher rowing performance (287.0 ± 17.7 W) compared to female rowers (180.9 ± 11.4 W). This difference is seen in both peak power output and relative peak power output per kilogram of body mass and per kilograms of muscle mass. With regard to peak power output, subelite traditional male rowers achieved 260 W [38], whereas mobile seat male and female rowers reached more than 400 W [42], and 278 ± 29 W [53], respectively. These differences in modalities can be explained by the longer leg range in mobile seat rowing, which achieves greater power output. Studies analysing the anthropometric characteristics of male and female rowers have shown the importance of anthropometric factors, such as body mass and height. These studies have established that body size and proportion play a crucial role in predicting rowing success [54,55]. As demonstrated in these studies and in the present research, male rowers have larger values of anthropometric characteristics and muscle mass. Furthermore, female rowers were found to have smaller lungs than males relative to body size and were more susceptible to arterial desaturation during intense exercise [56]. These findings suggest that differences in performance are due not only to body size or body composition but also to other physiological determinants [21]. In line with Mahler et al. [57], this study found that female rowers reached higher SR_max_ (37.9 ± 2.9 spm) than male rowers (33.9 ± 1.6 spm) and required a higher SR at VO_2max_. This is likely due to the relationship between intensity and stroke rate [58] as well as differences in body strength between females and male rowers [59].

The present study found that male rowers outperformed female rowers in terms of VO_2max_ and power output at VO_2max_. The male rowers achieved a VO_2max_ of 4.7 ± 0.5 l/min (62.1 ± 4.7 mL/kg/min) with a mean power of 280.0 ± 20.5 W, while the female rowers reached a VO2max of 3.2 ± 0.5 L/min (51.2 ± 6.6 mL/kg/min) with a mean power of 174.5 ± 12.9 W. In rowing, it is also appropriate to note VO_2max_ in both absolute values and ml/kg/min as boats support the rower’s weight [60]. French female mobile seat rowers showed 3.8 ± 0.3 L/min (52.4 ± 4.2 mL/kg/min) at a mean power of 266 ± 26 W [53], and female rowers of the National Collegiate Athletics Association in the EEUU obtained 3.8 ± 0.40 L/min [61]. Elite male rowers obtained VO_2max_ of 3.5 ± 0.1 L/min (53.3 ± 0.3 mL/kg/min) [57], while novice mobile seat rowers showed VO_2max_ of 4.1 ± 0.3 L/min (52.3 ± 3.3 mL/kg/min) at a mean power of 334.7 ± 20.2 W [42]. Generally, males have higher VO_2max_ than females, mainly due to higher systolic volume, higher haemoglobin concentration, higher amount of muscle mass, and lower amount of body fat [10,21,30,62]. Differences between female and male rowers seem consistent based on the anthropometric characteristics of the present study and taking into account that athletes with the largest body dimensions have the lowest VO_2max_ values expressed in relation to body mass [23]. According to Joyner [21], VO_2max_ values expressed as ml/kg/min are usually around 10% lower in females than in males. Regarding the ventilatory parameters, Ve at VO_2max_ of male rowers was 156.0 ± 17.2 L/min with Tv of 2.6 ± 0.3 L, while female rowers had Ve of 115.6 ± 11.9 L/min with Tv of 2.0 ± 0.2 L. According to Das et al., oxygen uptake and ventilation are closely related, with the increase in ventilation achieved through an increase in respiratory rate rather tidal volume [63]. VO_2_ shows a linear relationship with HR at submaximal intensities [64]. From 80-85% of VO_2max_, the relationship may not continue to be linear because HR may show a plateau at these intensities [42,65].

The results show a moderate correlation between VO_2max_ in mL/kg/min with performance in female (*r* = 0.36; *p* = 0.278) and male (*r* = 0.30; *p* = 0.399) traditional rowers. The results also show a moderate association between VO_2max_ in L/min with rowing performance in female rowers (*r* = 0.35; *p* = 0.297) but a strong correlation in male rowers (*r* = 0.69; *p* = 0.028). Bourdin et al. [53] also found a strong relationship between absolute VO_2max_ and rowing performance, but these results were obtained through a 2-km test in high-level female rowers rather than with a GXT. The fact that moderate correlations are not significant and strong correlations have small significant differences may be due to the sample size. It should be pointed out that while a high VO_2max_ may be a prerequisite for performance in endurance events at thigh level [66], the highest VO_2max_ value is not necessarily associated with the athlete having the best performance in an endurance test [62]. However, the relationships between VO_2max_ and performance used in the scientific literature have wide variations in the independent variables. When the range of each of these parameters is reduced, the correlations are reduced in magnitude or eliminated, suggesting that other variables also influence performance [62].

Considering the relative peak power output in watts per kilogram of body mass as a performance variable in rowing, the association with VO_2max_ in female rowers decreases (*r* = 0.24; *p* = 0.486), while the association in male rowers remains strong (*r* = 0.68; *p* = 0.031). This trend aligns with other studies of traditional rowing that found a strong correlation between male rowers’ body mass and rowing performance (*r* = 0.83; *p* < 0.05) [25]. Interestingly, the strongest correlation found in female rowers was between VO_2max_ and relative peak power output in watts per kilogram of muscle mass (*r* = 0.40; *p* = 0.228). These results are consistent with previous studies on body composition and traditional rowing performance that showed a very strong (*r* = 0.83; *p* < 0.05) and nearly perfect (*r* = 0.94; *p* < 0.05) correlation between muscle mass in kilograms and performance in female rowers [24,25].

The main limitation of this study is the limited sample size; thus, the results should be interpreted with caution. The individualized reference values established from this study’s findings are useful for developing training programs but should be considered in the context of each individual’s characteristics and needs. It is important to bear in mind that the evaluations were conducted on rowing ergometers rather than on boats with eight rowers, which could affect the studied parameters. As a portable gas analyser has been used, future research could be carried out directly on boats in water under controlled climatic conditions so that these do not interfere with the results. Since the portable gas analyser used was not equipped with a CO_2_ sensor, the parameters analysed were limited. In future research, a gas analyser with a CO_2_ sensor can be used to analyse the kinetics of the variables of this study at different ventilatory thresholds.

## 5. Conclusions

The results of this study show that male rowers outperform female rowers in performance tests, both in absolute values (in watts) and in relative values (in watts per kilogram of body mass and in watts per kilogram of muscle mass). Likewise, male rowers achieve higher maximal aerobic power with higher VO_2max_ values than female rowers in both absolute and relative terms. Ventilation and tidal volume values are also higher in male traditional rowers. A strong correlation between VO_2max_ (L/min) and rowing performance in watts was found in male rowers, while a moderate correlation was observed between VO_2max_ (mL/kg/min) and performance in watts per kilogram of body mass. In female rowers, the strongest relationships are between VO_2max_ (L/min) and performance in watts and VO_2max_ (mL/kg/min) and performance in watts per kilograms of body muscle. These values serve as a reference in the scientific literature for coaches and athletes in the assessment of performance and the comparison of internal load and external load values between highly trained male and female traditional rowers.

## Figures and Tables

**Table 2:** Mean values of maximal aerobic power and difference between female and male rowers.

	Female (*n* = 11)		Male (*n* = 10)		*p*	Effect Size	
	Mean ± SD	95% CI	Mean ± SD	95% CI		*d*	Size
Metabolic							
VO_2max_ (ml/kg/min)	51.2 ± 6.6	47.5–55.0	62.1 ± 4.7	58.9–64.8	<0.001 *	1.9	Large
VO_2max_ (l/min)	3.2 ± 0.5	2.9–3.4	4.7 ± 0.5	4.4–4.9	<0.001 *	3.2	Very Large
HR (bpm)	180.7 ± 9.1	175.7–185.4	185.9 ± 7.0	181.1–190.0	0.163	0.6	Small
Mechanical							
Power (W)	174.5 ± 12.9	167.3–181.8	280.0 ± 20.5	269.0–293.0	<0.001 *	6.2	Very Large
Power (W/kg)	2.8 ± 0.3	2.7–3.0	3.8 ± 0.3	3.6–4.0	<0.001 *	3.3	Very Large
Power (W/MMkg)	7.2 ± 0.4	6.9–7.4	8.4 ± 0.5	8.1–8.7	<0.001 *	2.7	Very Large
Time (s)	736.4 ± 71.5	698.2–779.9	1131.0 ± 127.3	1062.0–1209.0	<0.001 *	3.9	Very Large
SR (spm)	35.5 ± 4.0	33.3–37.7	32.9 ± 1.5	33.2–33.7	0.074	0.8	Moderate
Ventilatory							
Ve (l/min)	115.6 ± 11.9	109.2–122.5	156.0 ± 17.2	145.0–165.9	<0.001 *	2.8	Very Large
Tv (l)	2.0 ± 0.2	1.9–2.2	2.6 ± 0.3	2.5–2.8	<0.001 *	2.4	Very Large
Rf (breaths/min)	59.2 ± 7.4	55.1–63.5	60.4 ± 10.0	53.7–66.1	0.748	0.1	Trivial
Gas Exchange							
Ve/VO_2_	37.3 ± 6.1	34.3–40.9	33.5 ± 3.5	31.3–35.5	0.071	0.8	Moderate
FeO_2_ (%)	17.4 ± 0.6	17.1–17.7	17.1 ± 0.4	16.8–17.3	0.184	0.6	Small

SD: standard deviation; CI: confidence interval; VO_2max_: maximal oxygen consumption; HR: heart rate; bpm: beat per minute; SR: stroke rate; spm: stroke per minute; Rf: respiratory frequency; Tv: tidal volume; Ve: ventilation; FeO_2_: fraction of expired oxygen; *: statistically significance between female and male rowers (*p* < 0.05).

## Data Availability

The data presented in this study are available on reasonable request from the corresponding author.
